# A Narrative Review on Indian LGBTQ+ Community: Adapting Trauma Informed Care for this population

**DOI:** 10.1192/j.eurpsy.2026.11926

**Published:** 2026-07-31

**Authors:** V. Sharma, R. K. Suri, G. K. Suri, E. Ralhan, R. Sharma, S. Gunturu

**Affiliations:** 1 Dayanand Medical College & Hospital, Ludhiana, India; 2 Psychiatry, BronxCare Health System, The Bronx; 3 Psychology, University of British Columbia, Vancouver, United States; 4 Sharma’s Clinic for Mind & Braincare, Ludhiana, India

## Abstract

**Introduction:**

India has a complex relation with concepts of gender and sexuality. While a third gender or nature has been recognized since ancient time, India decriminalized homosexuality only in 2018, after waking up from its colonial hangover. Existing stigma in Indian society results in complex trauma and high psychiatric morbidity.

**Objectives:**

We explore the challenges faced, mental health struggles and resources available to the LGBTQ community through this narrative review. Additionally, we suggest adaptations to trauma and dissociation informed therapy to ideally support the Indian queer community.

**Methods:**

We searched PubMed using keywords “LGBT India mental health”, “LGBT Trauma psychotherapy”, and “LGBT India”, yielding 174 studies. Inclusion Criteria: relevant English original or meta-analytic studies from India. Exclusion criteria: non-English, non-Indian, inaccessible studies. After duplicate removal, abstract screening and full text analysis, a total of 37 studies (6 studies- LGBT Statistics, 13 studies- struggles of LGBT Indian community, 11 studies- mental health impact, 9 studies- available resources, and 7 studies- trauma and dissociation informed therapy) were included for this narrative review (Figure 1).

**Results:**

Familial, communal and police violence against the LGBTQ+ still exists in India. Stigma, microaggressions, homophobia, re-traumatization in healthcare causes complex PTSD and other psychiatric disorders (Figure 2). Government, non-profit and community-based interventions are lacking. Individually-tailored, socially-sensitive trauma-informed therapy would be ideal for the Indian queer community. ‘Narrative exposure therapy’ creating culturally empowering narratives, ‘Mentalization-based therapy’ reducing internalized stigma, ‘Prolonged exposure group therapy’ building a support system, ‘Milan family therapy’ combating negative stigma and ‘somatic therapy’ reclaiming one’s body, would help adapt existing western trauma and dissociation informed therapy models for the LGBTQ+ population in India (Figure 3).

**Image 1:**
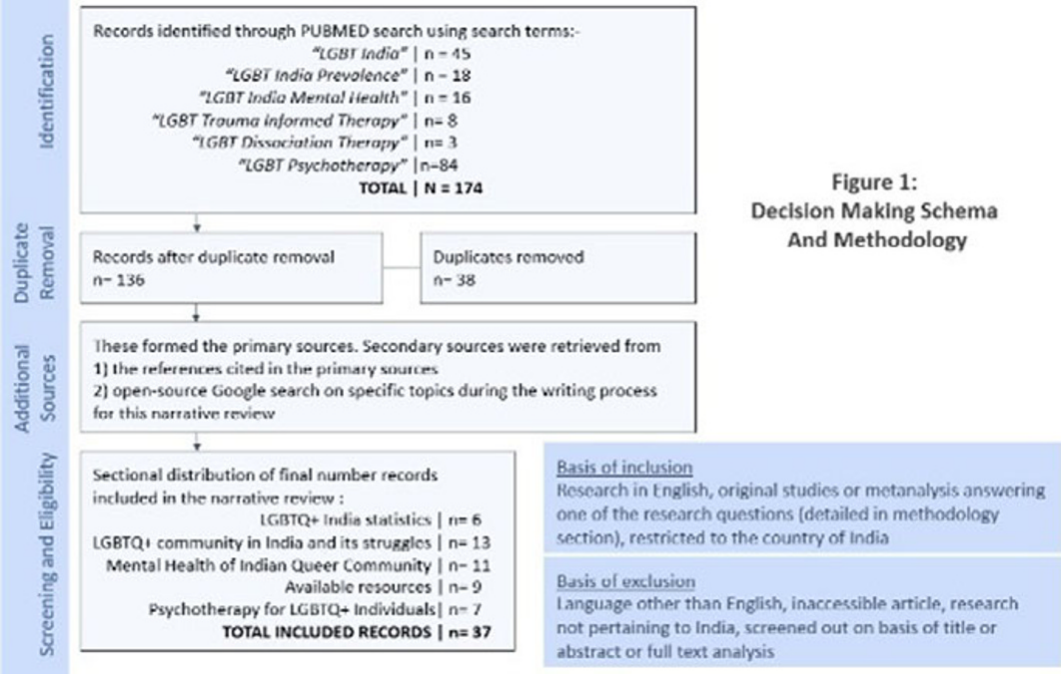
Image 1: Long description.

**Image 2:**
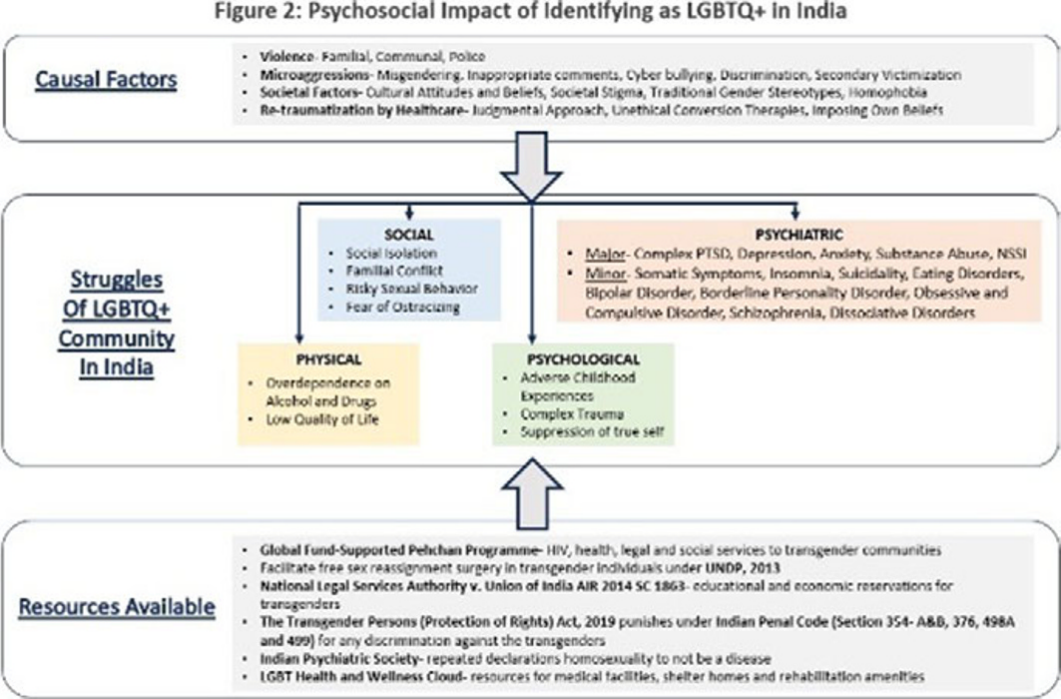
Image 2: Long description.

**Image 3:**
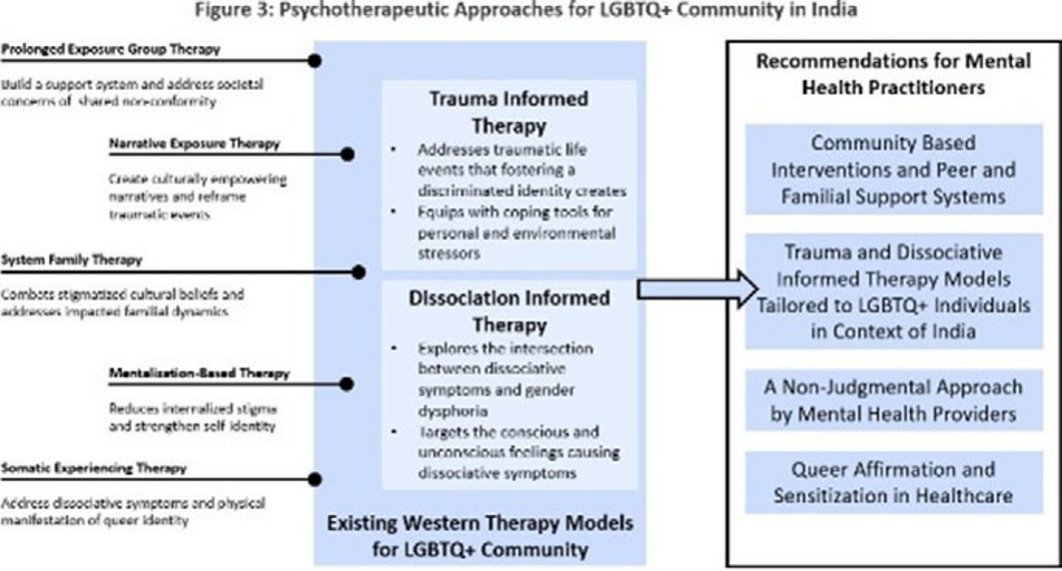
Image 3: Long description.

**Conclusions:**

By adapting a model of trauma informed therapy for the Indian LGBTQ+ community, we provide culturally and ethically sensitive recommendations for mental health professionals through this narrative review.

**Disclosure of Interest:**

None Declared

## Long descriptions

### Image 1: Long description

The flowchart is organized into four vertical stages labeled on the left side.

1. Identification. A top box lists records identified through P U B M E D search using specific terms. L G B T India n equals 45. L G B T India Prevalence n equals 18. L G B T India Mental Health n equals 16. L G B T Trauma informed Therapy n equals 8. L G B T Dissociation Therapy n equals 3. L G B T Psychotherapy n equals 84. The total n equals 174.

2. Duplicate Removal. An arrow points down to a split box. On the left, Records after duplicate removal n equals 136. On the right, Duplicates removed n equals 38.

3. Additional Sources. A box explains that primary sources were supplemented by secondary sources from references cited in primary sources and open-source Google searches.

4. Screening and Eligibility. The final stage consists of three boxes.

* The main box on the left shows the sectional distribution of the final 37 included records. L G B T Q plus India statistics n equals 6. L G B T Q plus community in India and its struggles n equals 13. Mental Health of Indian Queer Community n equals 11. Available resources n equals 9. Psychotherapy for L G B T Q plus Individuals n equals 7.

* A blue box on the right titled Basis of inclusion specifies research in English, original studies or metaanalysis answering research questions restricted to India.

* A second blue box on the right titled Basis of exclusion specifies languages other than English, inaccessible articles, research not pertaining to India, or records screened out based on title, abstract, or full text analysis.

Navigate back to Image 1:.

### Image 2: Long description

The flowchart is organized into three main horizontal sections.

Top Section: Causal Factors. A downward arrow points from this list to the central section. Factors include:

* Violence: Familial, Communal, Police.

* Microaggressions: Misgendering, Inappropriate comments, Cyber bullying, Discrimination, Secondary Victimization.

* Societal Factors: Cultural Attitudes and Beliefs, Societal Stigma, Traditional Gender Stereotypes, Homophobia.

* Re-traumatization by Healthcare: Judgmental Approach, Unethical Conversion Therapies, Imposing Own Beliefs.

Middle Section: Struggles Of L G B T Q plus Community In India. This section is divided into four sub-categories:

* Social (Top Left): Social Isolation, Familial Conflict, Risky Sexual Behavior, Fear of Ostracizing.

* Psychiatric (Top Right): Major includes Complex P T S D, Depression, Anxiety, Substance Abuse, N S S I. Minor includes Somatic Symptoms, Insomnia, Suicidality, Eating Disorders, Bipolar Disorder, Borderline Personality Disorder, Obsessive and Compulsive Disorder, Schizophrenia, Dissociative Disorders.

* Physical (Bottom Left): Overdependence on Alcohol and Drugs, Low Quality of Life.

* Psychological (Bottom Right): Adverse Childhood Experiences, Complex Trauma, Suppression of true self.

Bottom Section: Resources Available. An upward arrow points from this list to the central section. Resources include:

* Global Fund-Supported Pehchan Programme: H I V, health, legal and social services to transgender communities.

* Facilitate free sex reassignment surgery in transgender individuals under UNDP, 2013.

* National Legal Services Authority v. Union of India AIR 2014 SC 1863: educational and economic reservations for transgenders.

* The Transgender Persons (Protection of Rights) Act, 2019 punishes under Indian Penal Code (Section 354- A and B, 376, 498A and 499) for any discrimination against the transgenders.

* Indian Psychiatric Society: repeated declarations homosexuality to not be a disease.

* L G B T Health and Wellness Cloud: resources for medical facilities, shelter homes and rehabilitation amenities.

Navigate back to Image 2:.

### Image 3: Long description

The diagram is divided into three main sections from left to right.

1. Left Column (External Models): Five therapy types are listed with descriptions.

• Prolonged Exposure Group Therapy: Build a support system and address societal concerns of shared non-conformity.

• Narrative Exposure Therapy: Create culturally empowering narratives and reframe traumatic events.

• System Family Therapy: Combats stigmatized cultural beliefs and addresses impacted familial dynamics.

• Mentalization-Based Therapy: Reduces internalized stigma and strengthen self identity.

• Somatic Experiencing Therapy: Address dissociative symptoms and physical manifestation of queer identity.

2. Center Section (Existing Western Therapy Models for L G B T Q + Community): A blue box containing two primary categories.

• Trauma Informed Therapy: Addresses traumatic life events that fostering a discriminated identity creates; Equips with coping tools for personal and environmental stressors.

• Dissociation Informed Therapy: Explores the intersection between dissociative symptoms and gender dysphoria; Targets the conscious and unconscious feelings causing dissociative symptoms.

3. Right Column (Recommendations for Mental Health Practitioners): An arrow points from the center section to a white box containing four blue rectangular blocks.

• Community Based Interventions and Peer and Familial Support Systems.

• Trauma and Dissociative Informed Therapy Models Tailored to L G B T Q + Individuals in Context of India.

• A Non-Judgmental Approach by Mental Health Providers.

• Queer Affirmation and Sensitization in Healthcare.

Navigate back to Image 3:.

